# Genomic Occupancy of the Bromodomain Protein Bdf3 Is Dynamic during Differentiation of African Trypanosomes from Bloodstream to Procyclic Forms

**DOI:** 10.1128/msphere.00023-22

**Published:** 2022-06-01

**Authors:** Ethan Ashby, Lucinda Paddock, Hannah L. Betts, Jingwen Liao, Geneva Miller, Anya Porter, Lindsey M. Rollosson, Carrie Saada, Eric Tang, Serenity J. Wade, Johanna Hardin, Danae Schulz

**Affiliations:** a Department of Biology, Harvey Mudd College, Claremont, California, USA; b Department of Mathematics and Statistics, Pomona College, Claremont, California, USA; University of Texas Southwestern

**Keywords:** *Trypanosoma brucei*, chromatin, gene regulation, parasitology

## Abstract

Trypanosoma brucei, the causative agent of human and animal African trypanosomiasis, cycles between a mammalian host and a tsetse fly vector. The parasite undergoes huge changes in morphology and metabolism during adaptation to each host environment. These changes are reflected in the different transcriptomes of parasites living in each host. However, it remains unclear whether chromatin-interacting proteins help mediate these changes. Bromodomain proteins localize to transcription start sites in bloodstream parasites, but whether the localization of bromodomain proteins changes as parasites differentiate from bloodstream to insect stages remains unknown. To address this question, we performed cleavage under target and release using nuclease (CUT&RUN) against bromodomain protein 3 (Bdf3) in parasites differentiating from bloodstream to insect forms. We found that Bdf3 occupancy at most loci increased at 3 h following onset of differentiation and decreased thereafter. A number of sites with increased bromodomain protein occupancy lie proximal to genes with altered transcript levels during differentiation, such as procyclins, procyclin-associated genes, and invariant surface glycoproteins. Most Bdf3-occupied sites are observed throughout differentiation. However, one site appears *de novo* during differentiation and lies proximal to the procyclin gene locus housing genes essential for remodeling surface proteins following transition to the insect stage. These studies indicate that occupancy of chromatin-interacting proteins is dynamic during life cycle stage transitions and provide the groundwork for future studies on the effects of changes in bromodomain protein occupancy. Additionally, the adaptation of CUT&RUN for Trypanosoma brucei provides other researchers with an alternative to chromatin immunoprecipitation (ChIP).

**IMPORTANCE** The parasite Trypanosoma brucei is the causative agent of human and animal African trypanosomiasis (sleeping sickness). Trypanosomiasis, which affects humans and cattle, is fatal if untreated. Existing drugs have significant side effects. Thus, these parasites impose a significant human and economic burden in sub-Saharan Africa, where trypanosomiasis is endemic. T. brucei cycles between the mammalian host and a tsetse fly vector, and parasites undergo huge changes in morphology and metabolism to adapt to different hosts. Here, we show that DNA-interacting bromodomain protein 3 (Bdf3) shows changes in occupancy at its binding sites as parasites transition from the bloodstream to the insect stage. Additionally, a new binding site appears near the locus responsible for remodeling of parasite surface proteins during transition to the insect stage. Understanding the mechanisms behind host adaptation is important for understanding the life cycle of the parasite.

## INTRODUCTION

The ability to adapt to different environments is vital for parasites that live in different hosts. Trypanosoma brucei, the protozoan parasite that causes human and animal African trypanosomiasis, is one such organism. T. brucei lives in the bloodstream and tissues of the mammalian host (1, 2) and multiple organs within the tsetse fly vector as it travels from the gut to the salivary gland (3). Throughout its life cycle, the parasite adapts to each unique environment. Parasites living in the bloodstream of the mammal evade the host immune system through antigenic variation of surface proteins called variant surface glycoproteins (VSGs) (4, 5). Prior to transitioning to the fly, bloodstream parasites differentiate to stumpy forms that are transcriptionally preadapted for making the transition to the fly gut (6). Once the parasites arrive in the midgut, they differentiate fully to procyclic forms, and VSGs on the surface are replaced by procyclin proteins (7). Because the mammalian bloodstream and fly midgut differ in temperature, pH, and nutrient availability, the parasites undergo huge changes in morphology and metabolism. Underlying these changes are large differences in transcript levels for thousands of genes (8–11). Researchers have made great strides in understanding how environmental signals are sensed by the parasites and what signaling pathways may be important for the transition to stumpy and procyclic forms (12–18). It has also been demonstrated that RNA binding proteins play a role in differentiation processes (19–25). However, whether chromatin-interacting proteins play a role in initiating or regulating changes in transcript levels necessary for transition from the bloodstream to the procyclic stage in T. brucei is less well understood.

Experimental observations suggest that chromatin-interacting proteins might be involved in transcriptome reprogramming during the transition from bloodstream to procyclic forms. Notably, inhibition of chromatin-interacting bromodomain proteins in bloodstream parasites results in changes to the transcriptome that mirror those that occur as parasites transition from the bloodstream form to the procyclic form (26). Bromodomain proteins bind to acetylated histone tails in T. brucei and other model organisms (27–30) and have well established roles in gene regulation. Proteins with bromodomains have been shown to play a number of gene regulatory roles, including histone modification, chromatin remodeling, transcription factor recruitment, and enhancer or mediator complex assembly (31). They have been shown to be activators of gene transcription in some contexts, but can also be involved in gene silencing (32). Degradation of the mammalian Brd4 bromodomain protein in a leukemia cell line model results in a global disruption of productive transcription elongation driven by collapse of the elongation complex (33). Inhibition of bromodomain proteins in mammalian stem cells results in spontaneous differentiation (34, 35). Finally, mammalian Brd4 has been shown to be recruited to lineage-specific enhancers (36) and is necessary for adipogenesis and myogenesis (37). In T. brucei, seven bromodomain proteins (Bdfs) have been identified. Six of these proteins bind to transcription start sites at areas where polycistronic transcription units diverge (26, 29, 38). Members of the bromodomain protein family form distinct complexes in bloodstream forms (38). The focus of this study is Bdf3, which localizes to transcription start sites and has been shown to associate with Bdf5 and the histone acetyltransferase HAT2 (38). Bdf3 was chosen as a good first candidate because knockdown of this protein by RNA interference (RNAi) results in transcriptome changes similar to those that occur during differentiation from bloodstream to procyclic forms (26). While the localization of Bdf3 has been well characterized in bloodstream parasites, nothing is known about whether this localization is maintained during differentiation to procyclic forms. We hypothesized that Bdf3 might undergo changes in localization or occupancy at binding sites during differentiation from bloodstream to procyclic forms. Such changes in occupancy or localization could play a role in transcriptome reprogramming during differentiation.

To investigate whether localization of Bdf3 is dynamic during the transition from bloodstream to procyclic forms, we analyzed differentiating parasites using cleavage under targets and release using nuclease (CUT&RUN). This technique is an alternative to chromatin immunoprecipitation and sequencing (ChIP-seq) that avoids potential artifacts and can be performed in less time (39, 40). Our results indicate that the CUT&RUN protocol developed in mammalian systems can be modified for successful use in T. brucei. We were able to use data from differentiating parasites processed by CUT&RUN to show that 3 *de novo* sites of Bdf3 localization appear near the procyclin gene locus in differentiating parasites. More globally, occupancy at the majority of Bdf3 binding sites is transiently increased during the course of differentiation from bloodstream to procyclic forms.

## RESULTS AND DISCUSSION

### Optimization of CUT&RUN for bloodstream-form T. brucei.

The CUT&RUN protocol was originally developed for use in mammalian cell systems (39), so we set out to adapt the protocol to T. brucei bloodstream parasites. In brief, the CUT&RUN protocol works as follows. Cells are permeabilized and incubated with an antibody against the protein of interest. A fusion protein of protein A and micrococcal nuclease (pA-MN) is then added. The fusion protein binds to the antibody, and when calcium is added, the fusion protein cleaves the DNA immediately surrounding the protein of interest. The small DNA fragments that are released diffuse out of the nucleus and can be collected in the supernatant following centrifugation of the permeabilized cells. These small DNA fragments are used to generate a sequencing library, which can be analyzed with statistical methods already developed for ChIP-seq. When CUT&RUN is performed using an antibody against an abundant histone protein, a characteristic ladder of bands is produced that represent 150-bp increments corresponding to the size of DNA wrapped around a nucleosome (39, 41). Thus, we optimized the CUT&RUN protocol in the T. brucei EATRO1125 Antat 1.1 pleomorphic parasite line using an antibody against the abundant protein histone H3.

**FIG 1:**
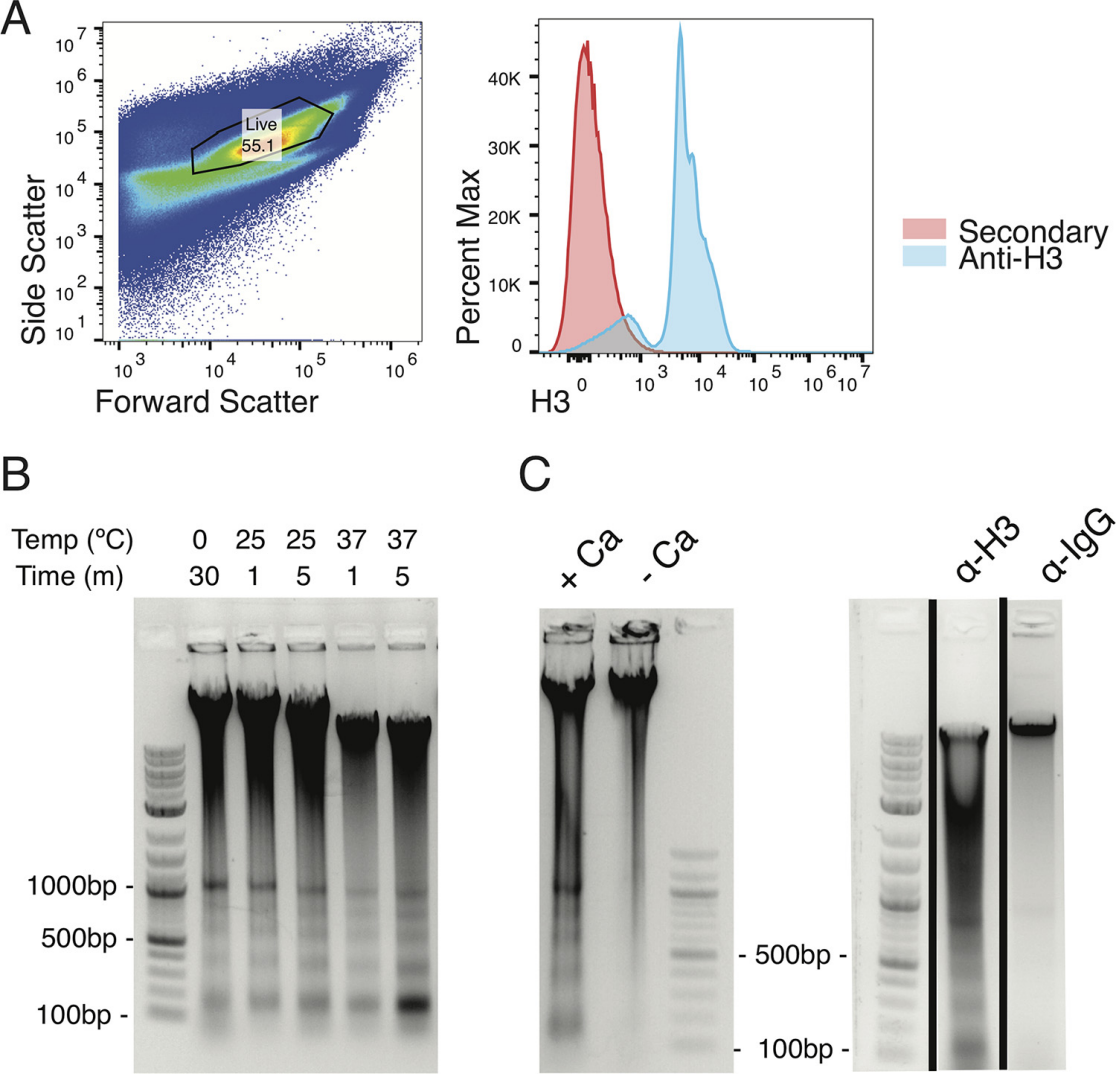
Optimization of CUT&RUN for T. brucei. (A) Flow cytometry analysis for parasites permeabilized with 0.1% saponin, incubated with rabbit anti-H3 primary antibody, and then stained with anti-rabbit antibody conjugated with PE. (Left) Forward and side scatter with the live gate shown. (Right) Live-gated parasites stained with anti-H3 (blue) or processed in parallel but with no anti-H3 antibody added and stained with secondary antibody alone (red). (B) Gel electrophoresis of DNA isolated from CUT&RUN-processed parasites using an anti-H3 antibody. CaCl_2_ was added for the indicated times and temperatures to activate micrococcal nuclease cutting. (C) Gel electrophoresis of DNA isolated from CUT&RUN-processed parasites using an anti-H3 antibody (left panels and first lane on right panel) or an IgG antibody (right panel, second lane). The left panel shows samples processed with or without CaCl_2_ to activate micrococcal nuclease cutting. The black lines indicate a gel cropped to show the indicated samples.

To optimize the permeabilization step of CUT&RUN, we developed a flow cytometry assay to test permeabilization of parasite membranes. Parasites were permeabilized with various detergents and then incubated with a primary antibody against histone H3. A fluorescently labeled secondary antibody was then added, and the parasites were assayed by flow cytometry. We observed anti-H3 staining following permeabilization with saponin, but did not see this staining in control samples where anti-H3 was not added (Fig. 1A). Having optimized the permeabilization conditions, we next tested a series of incubation times and temperatures for the protein A-micrococcal nuclease cleavage step of the protocol. We found that incubation for 5 min at 37°C most efficiently produced the characteristic ladder of bands expected following successful cleavage around histone H3 (Fig. 1B). However, because of the danger of nonspecific cleavage at 37°C (39), we processed experimental CUT&RUN samples for 5 min at 25°C. Targeted micrococcal nuclease cleavage was dependent on the addition of calcium and did not occur when a nonspecific control antibody was used (Fig. 1C). These results indicate that we successfully developed a CUT&RUN protocol for use in T. brucei bloodstream parasites.

### Bdf3-HA-tagged T. brucei parasites express a stumpy induction marker when grown to high density.

**FIG 2:**
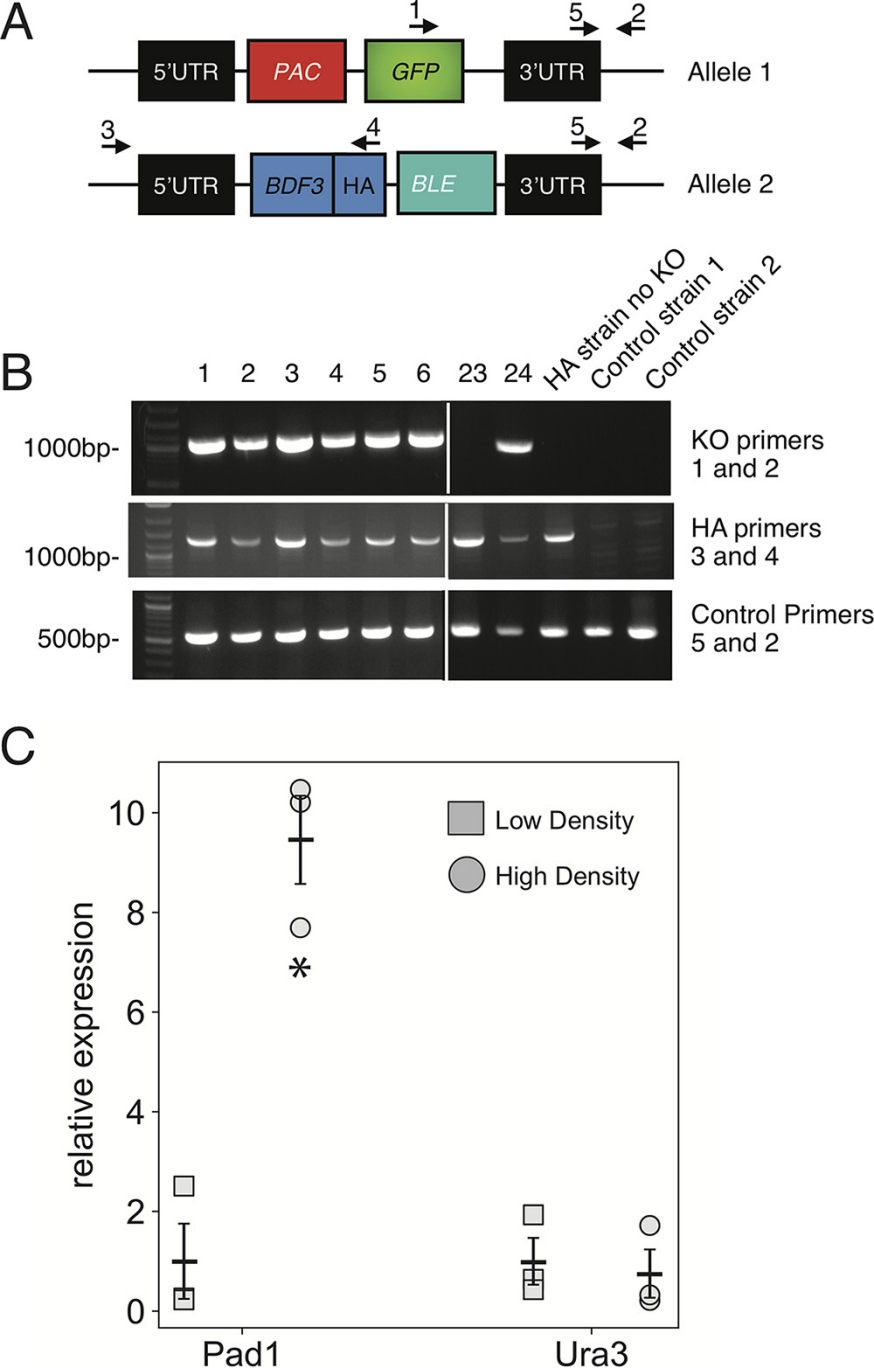
Generation of pleomorphic *BDF3*-HA/*BDF3* KO parasites. (A) Schematic of both endogenous *Bdf3* alleles following modification with a knockout construct (top) and an HA-tagging construct (bottom). Primers used to assay for correct integration are indicated. (B) Electrophoresis of genomic DNA amplified with primers to verify correct integration of the *BDF3* knockout construct (primers 1 and 2, top panel), primers to verify correct integration of the *BDF3*-HA tagging construct (primers 3 and 4, middle panel), and control primers that should amplify all genomic DNA samples (primers 5 and 2, bottom panel). Transformant clones are indicated as numbers. “HA strain no KO” indicates the strain modified with the HA construct but not the KO construct. The control strains are two nonpleomorphic strains used as negative controls for the modifications. The white line indicates cropping of the gel photo. (C) Quantitative PCR experiment to assay the transcript levels of *PAD1* and a control gene (*URA3*) in Bdf3-HA-tagged pleomorphic parasites at low density (less than 400,000 cells/mL) or high density (800 to 1,000,000 cells/mL). Values for the rRNA transcript were used for normalization. Each dot represents the average of 3 technical replicates; 3 biological replicates were used for the data shown here. The long horizontal bar represents the average value for the 3 biological replicates. Error bars represent the standard error. The data were scaled such that the average for the low-density samples was set to 1 to make it easier to compare different gene targets. * indicates a *P* value of <0.05 for a Student’s unpaired *t* test with equal variance comparing *Pad1* expression at low density versus high density.

To study the function of Bdf3 in differentiation-competent parasites, we generated EATRO1125 Antat 1.1 pleomorphic parasites with a hemagglutinin (HA)-tagged allele of *BDF3* and knocked out the remaining *BDF3* allele (Fig. 2A). Correct targeting to the endogenous *BDF3* locus was verified using a PCR assay (Fig. 2A and B), and our experiments were conducted with clone 6 of 24 tested clones. (For simplicity, some clones are omitted from the figure.) We tested our tagged parasites for their ability to generate stumpy forms by growing them to high density and measuring transcript levels of *PAD1*. We found increased transcript levels of *PAD1* when our Bdf3-HA-tagged parasites were grown to high density compared to parasites grown at low density (Fig. 2C). This accords with previous work showing increased *PAD1* transcript levels in pleomorphic parasites grown to high density (13) and indicates that our Bdf3-tagged pleomorphic strain shows the expected increase in the Pad1 stumpy induction marker once stumpy formation is induced.

### Bdf3 binding sites identified by CUT&RUN are similar to those identified by ChIP-seq in bloodstream parasites.

**FIG 3:**
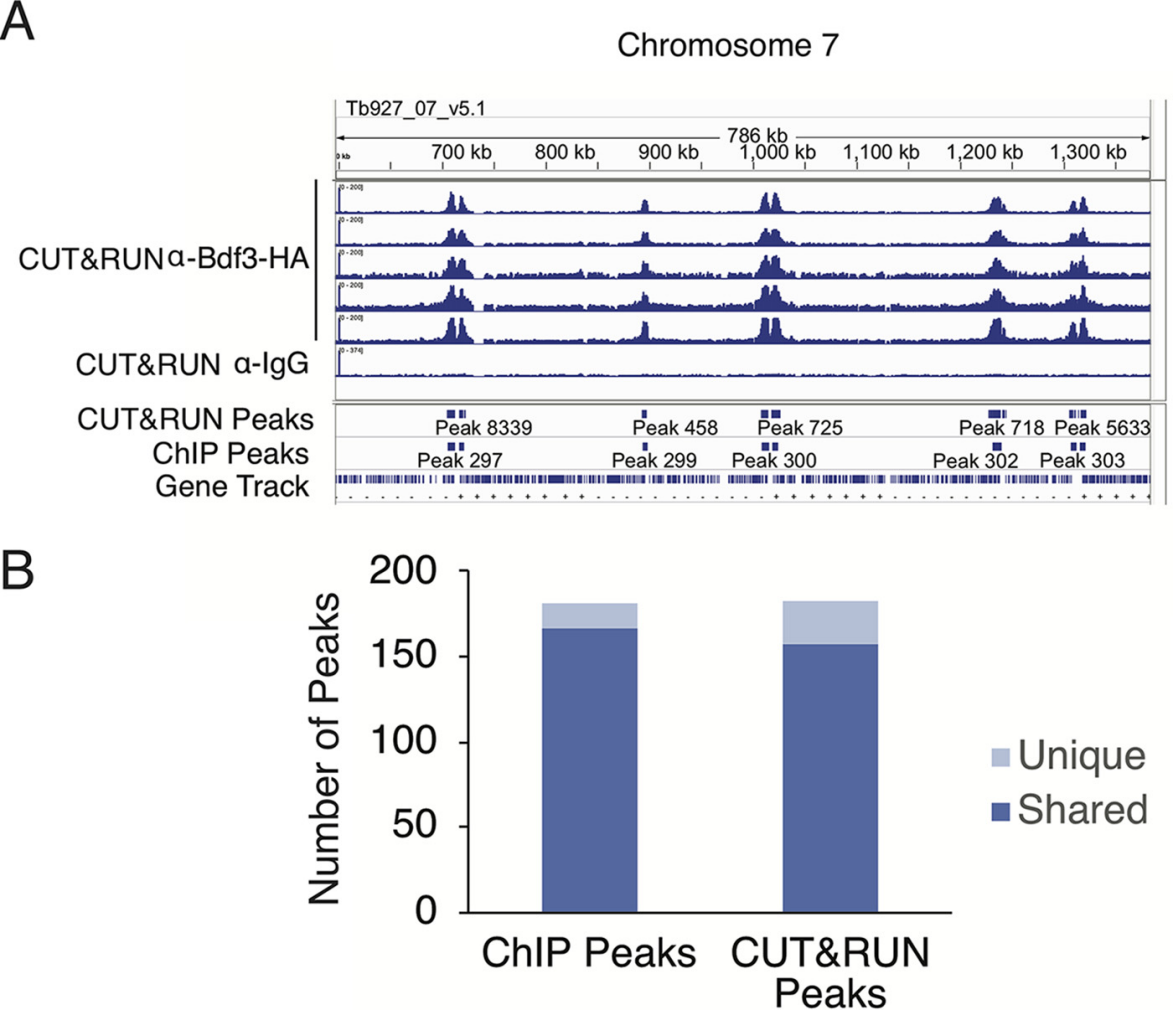
CUT&RUN-identified Bdf3 binding sites are similar to those previously found by ChIP-seq. (A) IGV display for a region of chromosome 7 showing sequencing tracks for five biological replicates processed for CUT&RUN using an anti-HA antibody in a pleomorphic Bdf3-HA-tagged parasite line. A control sample processed with anti-IgG is also shown. Blue boxes below sequencing tracks indicate peaks of Bdf3 localization called by MACS in CUT&RUN samples and in published Bdf3-HA ChIP experiments in monomorphic strains (26). The last row displays a gene track. (B) Plot showing the number of Bdf3 ChIP-identified peaks that do or do not overlap Bdf3 CUT&RUN-identified peaks and vice versa. Details of peak merging for this analysis are described in Materials and Methods.

Once we optimized the protocol for CUT&RUN in bloodstream parasites, we used the pleomorphic HA-tagged Bdf3 strain described above to perform CUT&RUN in bloodstream parasites using an anti-HA antibody. A nonspecific IgG antibody was used as a control. We used MACS software (42) to call peaks of Bdf3 localization, using the IgG sample as a control. We compared peaks found by CUT&RUN to our published Bdf3 peaks found using ChIP-seq (26) and found that peaks called by MACS for our CUT&RUN data set were very similar to peaks identified in our ChIP-seq data set (Fig. 3A). Ninety-two percent of peaks identified by ChIP-seq were also identified by CUT&RUN (Fig. 3B), and 86% of CUT&RUN peaks were identified by ChIP-seq. Overall, this suggests that CUT&RUN is a viable technique for producing localization data in T. brucei.

Because CUT&RUN does not use formaldehyde cross-linking, this technique could avoid artifacts that have been previously documented for ChIP-seq (40). In CUT&RUN, binding of the primary antibody to the target protein of interest occurs following permeabilization and prior to any other processing. The data produced for Bdf3 localization by CUT&RUN are quite similar to what has been seen previously for the localization of Bdf3 in bloodstream forms using ChIP-seq (Fig. 3B). Bdf3 was found to localize primarily to divergent strand switch regions thought to be transcription start sites. Seventy-six percent (112 out of 148) of all divergent strand switch regions (see Table S4 in the supplemental material) were within 5 kb of a Bdf3 peak. This is in accord with previous results that show Bdf3 localizing to regions where transcription is initiated (26, 29, 38). In addition, peaks of localization called by MACS showed substantial overlap between the two techniques. This is reassuring, as it suggests that data acquired by ChIP-seq represent physiological levels of binding for the T. brucei proteins that have been studied using the ChIP-seq technique. One situation where CUT&RUN might fail is in detecting transient protein-DNA interactions. For protein interactions of this type, a cross-linking-based technique might be necessary and/or preferable.

10.1128/msphere.00023-22.10 TABLE S4 Information on Bdf3 overlaps with genes and transcription start sites. Download Table S4, XLSX file, 0.3 MB. Copyright © 2022 Ashby et al. 2022 Ashby et al. https://creativecommons.org/licenses/by/4.0/ This content is distributed under the terms of the Creative Commons Attribution 4.0 International license.

Although the data produced by ChIP-seq and CUT&RUN are similar, CUT&RUN is faster. ChIP-seq requires at least two overnight steps to immunoprecipitate complexes and reverse the formaldehyde cross-linking, with a potential third overnight step to bind primary antibodies to beads. These steps are then followed by sequencing library processing. In contrast, CUT&RUN can be performed in ~4 h. We were able to perform CUT&RUN experiments successfully using 50 million cells, while use of 100 million cells is generally recommended for ChIP-seq protocols in T. brucei. It is possible that even fewer cells could be used successfully, but we did not formally test this. The originators of the protocol in mammalian cells claim that high-quality data can be obtained using 100 to 1,000 cells (43). The development of CUT&TAG (cleavage under targets and TAGmentation) has streamlined the process even further by eliminating some downstream steps associated with sequencing library preparation (43) and has further been refined to be fully automated (44). CUT&TAG has also been adapted for single-cell chromatin studies (45), which represents an exciting future application for the technique in T. brucei. A recent paper used single-cell sequencing to delineate transcriptome changes that occur during differentiation (8). The use of CUT&TAG on single cells during differentiation might delineate the accompanying changes in occupancy for chromatin-associated proteins throughout the differentiation process. Excitingly, CUT&RUN has been successfully adapted for use in Toxoplasma gondii to identify a master regulator of differentiation (46).

### Bdf3 localizes to a region near the procyclin gene locus following differentiation.

**FIG 4:**
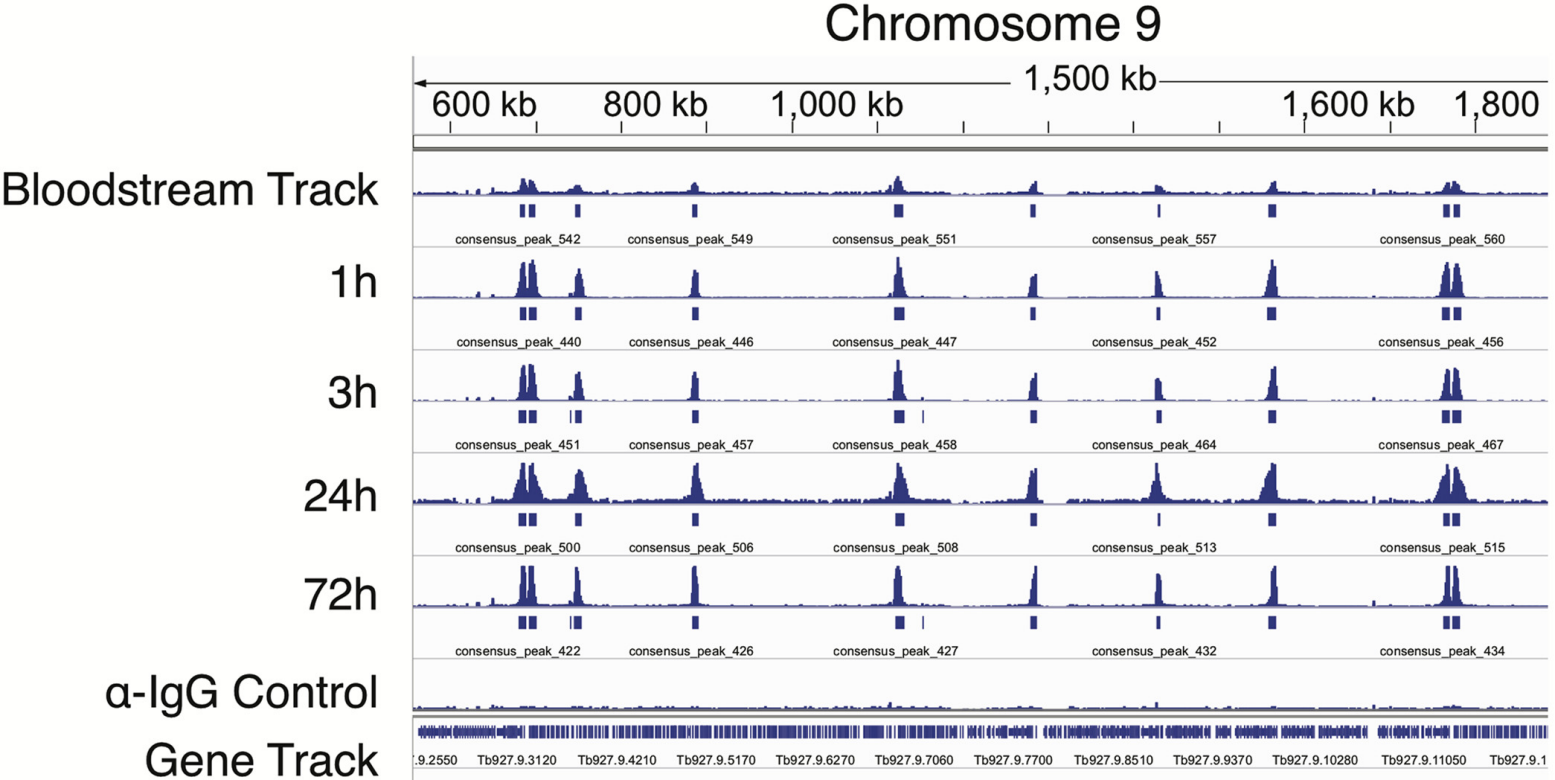
Most Bdf3 binding sites are retained throughout differentiation. Shown is the IGV display for a region of chromosome 9 showing sequencing tracks for overlaid biological replicates processed for CUT&RUN using an anti-HA antibody in a pleomorphic Bdf3-HA-tagged parasite line. A control sample processed with anti-IgG is also shown. Solid blue boxes below each sequencing track indicate peaks of Bdf3 localization identified by MACS.

In order to ascertain whether the localization of Bdf3 is altered as parasites differentiate from the bloodstream to the procyclic form, we induced differentiation of our pleomorphic Bdf3-HA-tagged strain by resuspending parasites in differentiation medium, adding 6 mM *cis*-aconitate, and incubating them at 27°C. We harvested parasites in triplicate at 1 h, 3 h, 24 h, and 72 h postdifferentiation and performed CUT&RUN using an anti-HA antibody. Bloodstream parasites were also processed the same way using 5 biological replicates. Sequencing libraries were generated, and the resulting reads were trimmed for quality and aligned to the T. brucei genome. MACS (42) was then used to call peaks of Bdf3 localization at every time point (Fig. 4; peak locations given in Tables S1 and S3). Visual inspection of the results revealed that almost all Bdf3 peaks identified in all 5 replicates of bloodstream parasites were also identified as peaks at every time point thereafter (Fig. 4).

10.1128/msphere.00023-22.7 TABLE S1 Diffbind normalized tag counts over time for Bdf3 peaks identified by MACS. Normalization is performed 4 different ways, and the normalization method is indicated by the tab name in the Excel document. Consensus sites for Bdf3 binding regions for all time points are provided in a separate tab. Download Table S1, XLSX file, 0.7 MB. Copyright © 2022 Ashby et al. 2022 Ashby et al. https://creativecommons.org/licenses/by/4.0/ This content is distributed under the terms of the Creative Commons Attribution 4.0 International license.

10.1128/msphere.00023-22.9 TABLE S3 Normalized tag counts over time for Bdf3 sites with dynamic occupancy identified by DiffBind using four normalization methods. Normalized tag counts over time for control regions that are not Bdf3 binding sites are provided in a separate tab. Download Table S3, XLSX file, 0.5 MB. Copyright © 2022 Ashby et al. 2022 Ashby et al. https://creativecommons.org/licenses/by/4.0/ This content is distributed under the terms of the Creative Commons Attribution 4.0 International license.

**FIG 5:**
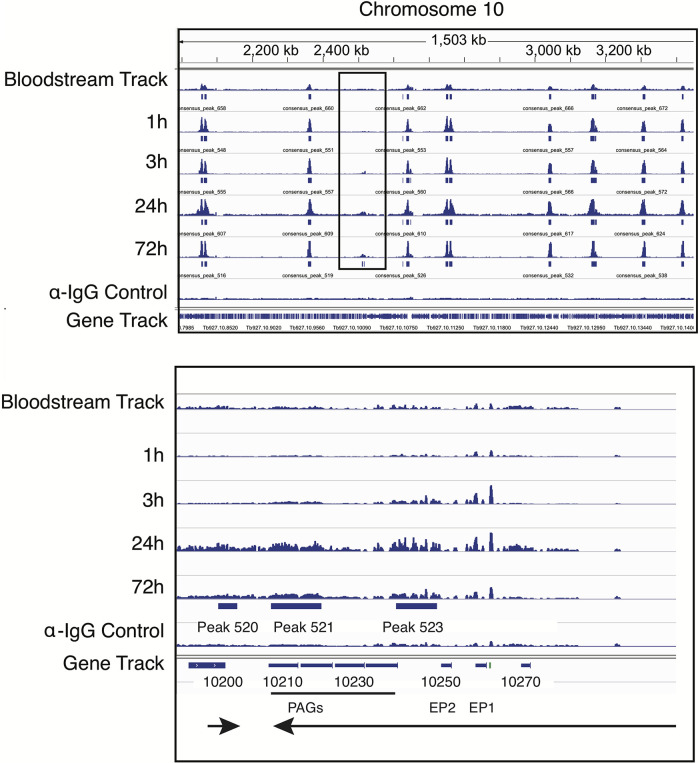
*De novo* Bdf3 peaks appear at the *EP1* locus after differentiation to the procyclic form is induced. (Top) IGV display for a region of chromosome 10 showing sequencing tracks for overlaid biological replicates processed for CUT&RUN using an anti-HA antibody in a pleomorphic Bdf3-HA-tagged parasite line. A control sample processed with anti-IgG antibody is also shown. Solid blue boxes below each sequencing track indicate peaks of Bdf3 localization identified by MACS. The black rectangle outlines the location of a new peak forming at the *EP1* locus. (Bottom) Same as the top panel, except zoomed in on the *EP1* locus for chromosome 10. Three peaks are called at 72 h that did not appear previously. The solid green box in front of *EP1* indicates the promoter. Arrowheads indicate the direction of transcription.

While most MACS-identified Bdf3 peaks were retained throughout the course of differentiation, one notable exception to this trend was found at chromosome 10 in the region of the procyclin gene locus (47). MACS called three Bdf3 peaks in this region at 76 h postdifferentiation that were not identified as peaks earlier in the time course (Fig. 5). Quantification of Bdf3 occupancy at this site using the DiffBind program (48) revealed that Bdf3 occupancy increases at 3 h and remains high throughout the remainder of the time course (Fig. S1A) We tested *EP1* transcript levels following differentiation in our pleomorphic line using the same time points and found an ~5-fold increase in *EP1* transcript levels at 1 h and an ~20- to 50-fold increase at 3 h (Fig. S2). While the MACS algorithm identified Bdf3 peaks at 3 specific sites (indicated by the peaks labeled 520, 521, and 523), the gene track for Bdf3 shows widespread binding over a large region at this locus (Fig. 5B). This is especially interesting because transcripts from the *EP* and *PAG* genes near this locus are increased during the transition from bloodstream to procyclic forms so that parasites can remodel their VSG surface coat with procyclin. In contrast to many genes driven by polymerase II (Pol II), procyclin genes are instead transcribed by Pol I (49–52). Additionally, while Pol II-driven genes are thought to be largely regulated posttranscriptionally, procyclin genes have been shown to be transcriptionally regulated (53). While RNA binding proteins have been shown to have a role in stabilizing transcripts of procyclin genes (23–25), the *de novo* localization of Bdf3 to this region may indicate that bromodomain proteins play a role in inducing or maintaining increased levels of procyclin transcripts during differentiation (Fig. 5). This idea is in accord with findings that chromosomal context is important for procyclin promoter regulation (54). This would be interesting to test in future studies: perhaps through the use of a tethering experiment in which Bdf3 is artificially localized to the *EP* locus in bloodstream forms. An increase in the level of transcript for *EP1* following artificial tethering would support a model in which the *de novo* appearance of Bdf3 at this locus helps to increase transcript levels of procyclin genes nearby, facilitating surface remodeling of the parasite.

10.1128/msphere.00023-22.1 FIG S1 The *EP* locus shows a different pattern of occupancy compared to loci within 5 kb of differentiation-associated genes. (A) Plot showing log_2_ RLE normalized counts for Bdf3 occupancy at the 3 *de novo* peaks detected at the *EP* locus. The plot in the right panel includes individual values for each replicate. (B) Plot showing log_2_ RLE normalized counts for Bdf3 occupancy at sites within 5 kb of genes known to be associated with differentiation. Download FIG S1, TIFF file, 1.1 MB. Copyright © 2022 Ashby et al. 2022 Ashby et al. https://creativecommons.org/licenses/by/4.0/ This content is distributed under the terms of the Creative Commons Attribution 4.0 International license.

10.1128/msphere.00023-22.2 FIG S2 *EP1* expression increases at 3 h postdifferentiation in pleomorphic parasites. (A) Quantitative PCR experiment to assay the transcript levels of *EP1* and a control gene (*SF1*) in Antat 1.1 pleomorphic parasites following differentiation at the indicated time points. Values for the rRNA transcript were used for normalization. Each dot represents the average of 3 technical replicates; 3 biological replicates were used for the data shown here. The horizontal bar represents the average value for the 3 biological replicates. Error bars represent the standard error. The data were scaled such that the average for the 0-h samples was set to 1 to make it easier to compare different gene targets. * indicates a *P* value of <0.05 for a Student’s unpaired *t* test with equal variance comparing *EP1* expression at the indicated time point versus the 0-h time point. (B) Same as in panel A, except using the Bdf3-HA-tagged pleomorphic line. Each dot represents the average of 3 technical replicates; 3 biological replicates were used for the data shown here. The horizontal bar represents the average value for the 3 biological replicates. Error bars represent the standard error. The data were scaled such that the average for the 0-h samples was set to 1 to make it easier to compare different gene targets. Download FIG S2, TIF file, 0.4 MB. Copyright © 2022 Ashby et al. 2022 Ashby et al. https://creativecommons.org/licenses/by/4.0/ This content is distributed under the terms of the Creative Commons Attribution 4.0 International license.

Custom scripts were used to systematically check for other instances of *de novo* Bdf3 peak formation following differentiation. While a number of sites were identified, visual inspection revealed that most of these sites were likely false positives. Many were in regions with high background in the IgG control samples. Thus, it is likely that for some time points, these regions just made it over the threshold to be called by MACS as a peak, while at other time points, they did not, leading to a false-positive result. Table S2 lists each of the sites with a numerical code given by visual inspection, with 0 indicating no evidence of *de novo* peak formation by visual inspection, 1 indicating some evidence, and 2 indicating good evidence. Sites that received a score of 1 include the *GPEET* (Tb927.6.510) procyclin locus on chromosome 6 (Fig. S6) and the invariant surface glycoprotein (*ISG75*) locus on chromosome 5 proximal to Tb927.5.360 (Fig. S3). Both of the protein products for these loci are associated with differentiation processes. A similar analysis was performed to check for MACS-called Bdf3 peaks present in bloodstream forms that were not present at subsequent time points. Again, many of these loci likely represent false positives, and only two sites were scored as 1: a *VSG* locus on chromosome 5 and an *ESAG* locus on chromosome 1 (Table S2).

10.1128/msphere.00023-22.3 FIG S3 IGV display for a region of chromosome 5 showing sequencing tracks for overlaid biological replicates processed for CUT&RUN using an anti-HA antibody in a pleomorphic Bdf3-HA tagged parasite line. A control sample processed with anti-IgG is also shown. Solid blue boxes below each sequencing track indicate peaks of Bdf3 localization identified by MACS. (A) *De novo* peak at a locus near a putative *ISG* (Tb927.5.309b); (B) *de novo* peak near a cluster of *ISG75* genes (Tb927.5.350-380). Download FIG S3, TIF file, 1.2 MB. Copyright © 2022 Ashby et al. 2022 Ashby et al. https://creativecommons.org/licenses/by/4.0/ This content is distributed under the terms of the Creative Commons Attribution 4.0 International license.

10.1128/msphere.00023-22.6 FIG S6 IGV display for a region of chromosome 6 at the *GPEET* (Tb927.6.530) locus showing sequencing tracks for overlaid biological replicates processed for CUT&RUN using an anti-HA antibody in a pleomorphic Bdf3-HA-tagged parasite line. A control sample processed with anti-IgG is also shown. Blue boxes below each sequencing track indicate peaks of Bdf3 localization identified by MACS. Download FIG S6, TIF file, 0.4 MB. Copyright © 2022 Ashby et al. 2022 Ashby et al. https://creativecommons.org/licenses/by/4.0/ This content is distributed under the terms of the Creative Commons Attribution 4.0 International license.

10.1128/msphere.00023-22.8 TABLE S2 Analysis of Bdf3 binding sites that appear and disappear during differentiation. Download Table S2, XLSX file, 0.01 MB. Copyright © 2022 Ashby et al. 2022 Ashby et al. https://creativecommons.org/licenses/by/4.0/ This content is distributed under the terms of the Creative Commons Attribution 4.0 International license.

### Occupancy of Bdf3 at genomic binding sites transiently increases as parasites differentiate from bloodstream to procyclic forms.

Having ascertained that most sites of Bdf3 localization are retained throughout differentiation, we next wanted to measure whether occupancy at these sites is altered by quantifying tag counts within each Bdf3 peak over time. To do so, we took advantage of the DiffBind program (48, 55), which is designed to identify changes in protein occupancy under different conditions. DiffBind takes BAM files and MACS-called peaks as input and first finds consensus peaks that are present in all biological replicates using the provided MACS files. These consensus peaks are considered regions of interest. The program normalizes the data and computes a normalized tag count for regions of interest using a 400-bp region surrounding the peak summit. A region is considered to have a change in occupancy if there is a statistically significant change in tag count for the region around the peak summit at a time point after differentiation compared to the bloodstream samples.

**FIG 6:**
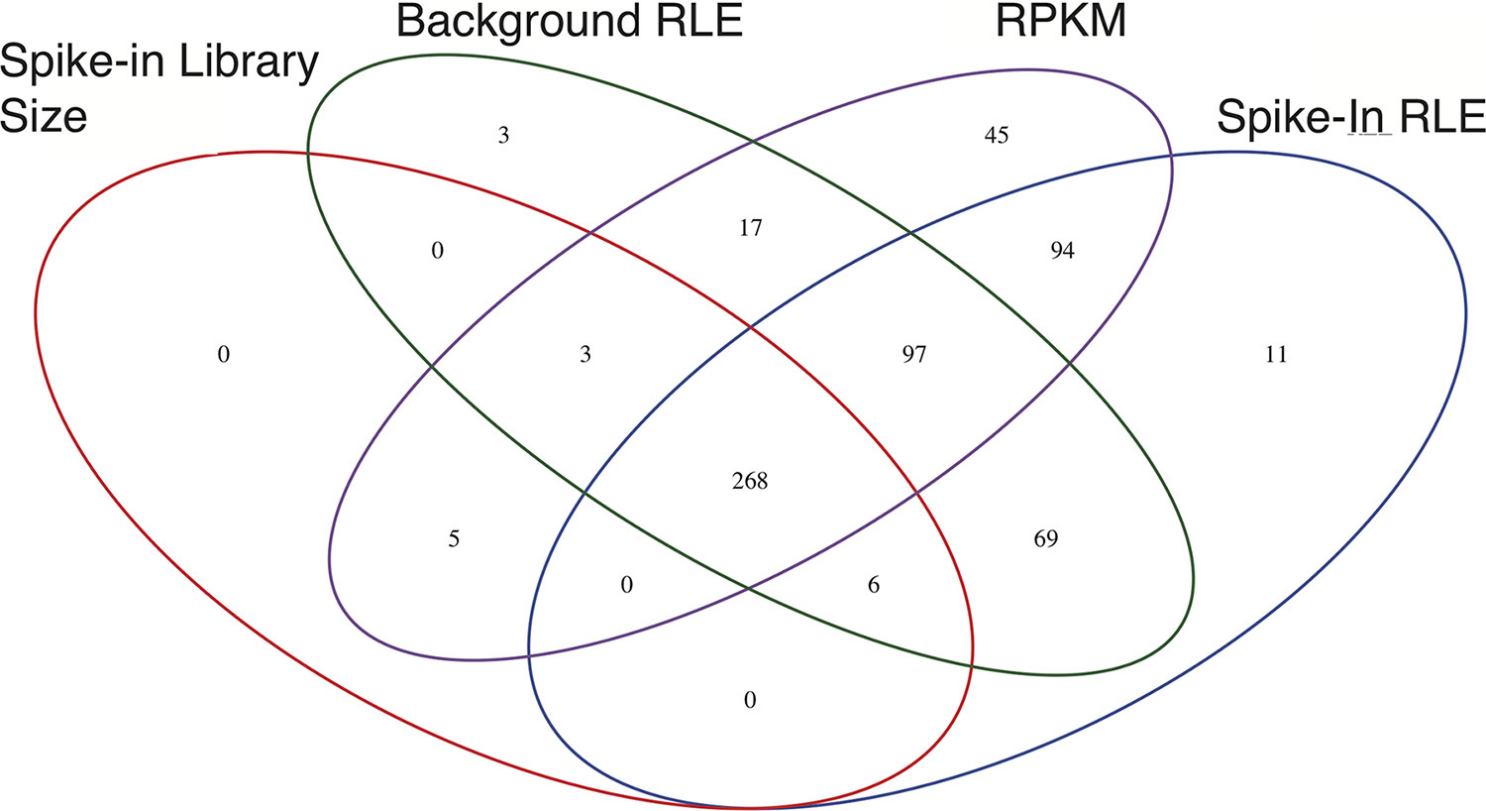
Changes in occupancy for Bdf3 are found at many loci throughout the genome following induction of differentiation from the bloodstream to the procyclic form. A Venn diagram shows the number of sites identified by DiffBind as having a statistically significant change in occupancy compared to bloodstream samples by four different normalization methods.

The method of normalization has been shown to have an outsized effect on whether a particular region is identified as having a significant change in occupancy (48). To circumvent this potential issue, we used four different normalization methods to identify regions with a change in occupancy over the course of differentiation. The regions identified as having a change in occupancy by all four normalization methods are considered “high-confidence” differentially occupied regions. The normalization methods used include (i) reads per kilobase of transcript per million mapped reads (RPKM), (ii) spike-in library size normalization, which normalizes using spiked-in yeast DNA for each sample, (iii) background relative log expression (RLE), where counts are divided by sample-specific size factors determined by the median ratio of gene counts relative to geometric mean per gene (a method similar to DESeq), and (iv) background RLE of spiked-in reads, which uses the former method with spiked-in yeast in reads. In total, we found 268 “high-confidence” regions that were identified as having a significant change in occupancy at a time point following differentiation compared to the bloodstream data (Fig. 6).

**FIG 7:**
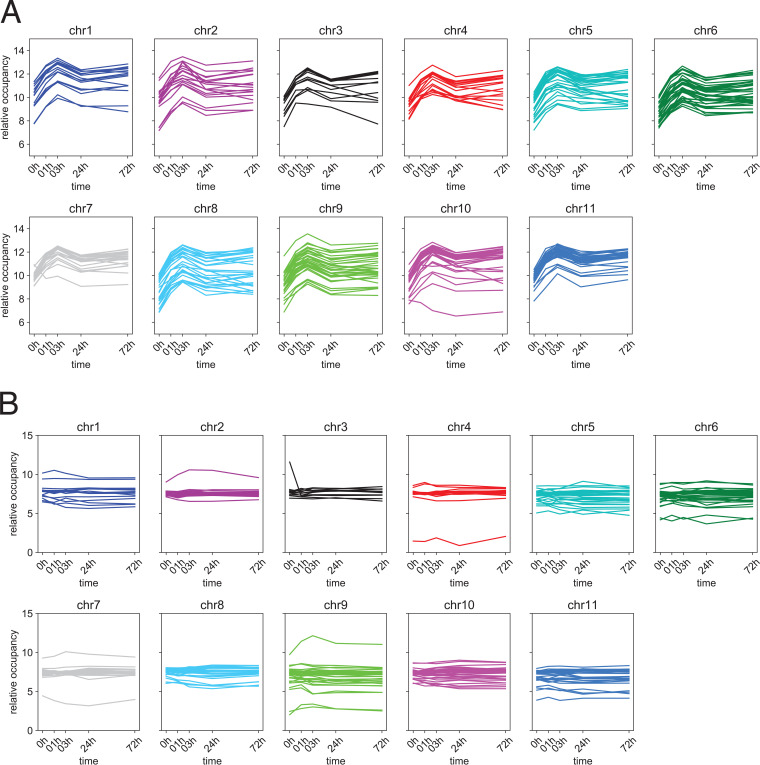
Bdf3 occupancy peaks at 3 h after induction of differentiation. (A) Plots of log_2_ normalized tag counts over time for each “high-confidence” Bdf3 binding site with a statistically significant change in occupancy after induction of differentiation to the procyclic form using background RLE normalization and arrayed by chromosome. (B) Plots of log_2_ normalized tag counts over time for control regions that are not identified as Bdf3 binding sites using background RLE normalization.

We plotted the normalized tag counts for each of the 268 “high-confidence” differentially occupied Bdf3 binding sites arrayed by chromosome (Fig. 7A; Fig. S4 and Table S3). There was a remarkable similarity to the patterns of occupancy for these differentially bound regions over time, with most regions showing a peak in occupancy at 3 h postdifferentiation and a decrease in occupancy thereafter. In order to ensure that the peak in occupancy observed for Bdf3 at 3 h postdifferentiation was not an artifact, we randomly shuffled Bdf3 binding site locations using bedtools (56). The regions were shuffled such that each shuffled site was retained on the same chromosome as the original site, and all regions identified as consensus sites were excluded. These shuffled regions were then run through the DiffBind program and normalized the same way as the Bdf3 consensus sites (Fig. 7B; Fig. S5 and Table S3). When tag counts for shuffled control regions were plotted over time, we did not observe the same peak in the number of tag counts at the 3-h time point, indicating that the peak in Bdf3 occupancy that occurs 3 h after differentiation represents a genuine change in the amount of Bdf3 found at that location, at either the single-cell or the population level. Interestingly, the occupancy for peaks at the *EP* locus does not show this pattern. Instead, occupancy shows a pronounced increase at 3 h and remains high for the duration of the time course (Fig. S1). The fact that there is a pronounced increase in *EP1* expression at 3 h (Fig. S2) may indicate that the *de novo* formation of Bdf3 peaks at this time point could help increase transcript levels of *EP1* at this locus. While the MACS algorithm did not call Bdf3 peaks at this locus until 72 h, quantification by Diffbind as well as visual inspection of the sequencing tracks indicate that the peaks may be forming as early as 1 to 3 h (Fig. 5; Fig. S1).

10.1128/msphere.00023-22.4 FIG S4 Plots of log_2_ normalized tag counts over time for each “high-confidence” Bdf3 binding site with a statistically significant change in occupancy after induction of differentiation to the procyclic form using indicated normalization. Download FIG S4, TIFF file, 1.6 MB. Copyright © 2022 Ashby et al. 2022 Ashby et al. https://creativecommons.org/licenses/by/4.0/ This content is distributed under the terms of the Creative Commons Attribution 4.0 International license.

10.1128/msphere.00023-22.5 FIG S5 Plots of log_2_ normalized tag counts over time for control regions that are not identified as Bdf3 binding sites using indicated normalization. Download FIG S5, TIFF file, 1.2 MB. Copyright © 2022 Ashby et al. 2022 Ashby et al. https://creativecommons.org/licenses/by/4.0/ This content is distributed under the terms of the Creative Commons Attribution 4.0 International license.

The fact that the peak of Bdf3 occupancy is reached 3 h following addition of *cis*-aconitate is especially interesting in light of work showing that commitment to differentiation occurs 2 to 3 h after *cis*-aconitate treatment (57). This group further demonstrated that protein synthesis is required to generate “memory” of exposure to the differentiation signal. One possible model is that bromodomain protein occupancy at transcription start sites facilitates commitment at 3 h following differentiation by an unknown mechanism. For instance, if increased occupancy led to an increase in transcript levels, this could aid in the protein synthesis required for commitment to differentiation.

Of the 268 “high-confidence” sites with changes in Bdf3 occupancy, 162 are within 5 kb of a divergent strand switch region considered to be a site of transcription initiation in T. brucei. This might be an underestimate of the true number of dynamically occupied Bdf3 sites at divergent strand switch regions because we used only unique alignments, and thus we lack data for highly repetitive regions with divergent strand switch regions near the ends of the chromosomes. Of 148 divergent strand switch regions we examined, 60% were within 5 kb of a “high-confidence” dynamically occupied Bdf3 site. The effect of the increased occupancy at sites of Bdf3 localization is not yet known for T. brucei, but in other systems, changes in occupancy for chromatin binding proteins can reflect an underlying change in particular histone tail modifications that are bound by the chromatin binding protein (55). In our particular case, a change in histone acetylation at transcription start sites could result in the observed increase in Bdf3 occupancy at these sites as parasites begin differentiation. Future studies could investigate the acetylation levels at transcription start sites after the induction of differentiation from the bloodstream to the procyclic form. A transient increase in acetylation at transcription start sites might result in a corresponding transient increase in bromodomain protein localization at these sites that is observed for Bdf3 (Fig. 7). This leaves us with the question of how an increase in acetylation might support the differentiation process. One model is that such an increase in acetylation might increase genomic transcription overall and thus help the parasites exit the relatively quiescent cell-cycle-arrested stumpy stage and transition to the dividing procyclic stage. In other systems, quiescence results in a global decrease in acetylation levels (58–60). Specific chromatin remodeling enzymes are required to generate hypertranscription necessary for exiting the quiescent state (61). If an increase in acetylation levels and increased transcription are necessary during the transition from the quiescent stumpy form to the cycling procyclic form, this could result in the observed increase in Bdf3 occupancy following the differentiation cue. Bromodomain protein-mediated regulation of global Pol II transcript levels has been demonstrated in *Leishmania* (62). Transcriptome sequencing (RNA-seq) analyses are typically normalized in such a way as to obscure global increases or decreases in transcript levels (63). Thus, it might be interesting to examine whether the exit from the stumpy to the procyclic stage results in a global increase in transcript levels for Pol II-regulated genes.

Bromodomain inhibition in bloodstream parasites using the small molecule inhibitor I-BET151 results in changes in the transcriptome that mirror those that occur during differentiation from the bloodstream to the procyclic stage in many ways, including an increase in *EP1* transcript levels (26). We observed a sharp decrease in bromodomain protein occupancy for at least 268 Bdf3 binding sites in the period between 3 h and 24 h after the initiation of differentiation (Fig. 7). One model for why bromodomain inhibition may trigger transcriptome changes akin to differentiation is that binding of the drug to its bromodomain protein target causes a sharp decrease in occupancy for bromodomain proteins in bloodstream forms, as is seen at 3 h postdifferentiation (Fig. 7). We did observe decreased enrichment of Bdf3 in I-BET151-treated bloodstream parasites at several sites, consistent with this model (26). If the decrease in Bdf3 occupancy in differentiating parasites is partly responsible for promoting the transition to a procyclic-specific transcriptome, then an artificial I-BET151-induced decrease in Bdf3 occupancy might also produce the observed changes in transcript levels for procyclic-associated genes.

An unanswered question is whether the transient increase in Bdf3 occupancy observed at Pol II initiation sites during differentiation induced by *cis*-aconitate and temperature shift is also observed during the I-BET151/Bdf3 knockdown-induced differentiation-like phenotype. The earliest time point that was used to check for whether I-BET151 decreased binding to Bdf3 targets was 6 h, and thus a transient increase in occupancy followed by I-BET151 treatment (by some unknown mechanism) might have been missed. Additionally, Bdf3 target occupancy was not tested genome-wide in that previous study (26). It is also possible that the I-BET151-induced differentiation phenotype occurs via an entirely different mechanism than the one induced by *cis*-aconitate and temperature shift and that this mechanism does not require a transient increase in Bdf3 occupancy. One model is that the I-BET151 disruption of monoallelic expression is responsible for driving the transcriptome changes that are observed following drug treatment, consistent with studies by Batram et al. (64). The idea that different triggers can result in differentiation phenotypes is also supported by other work (65, 66).

We previously observed an increase in *EP1* transcript levels following bromodomain inhibition (26). This may indicate that Bdf3 occupancy at the *EP1* locus is not required to increase transcript levels of *EP1* or that the increase in *EP1* transcript levels occurs via a different mechanism following bromodomain inhibition, as mentioned above. Alternatively, residual levels of bromodomain protein following inhibition may have been sufficient to drive the observed increase in *EP1.* While isothermal titration calorimetry (ITC) experiments confirmed binding of I-BET151 to trypanosome bromodomains (26), it is also possible that I-BET151 has off-target effects that result in the observed increase in *EP1* transcript levels.

A number of genes known to be associated with differentiation were within 5 kb of a dynamically occupied Bdf3 site. Table S4 lists all genes within 5 kb of “high-confidence” Bdf3 sites and separates out those identified by Queiroz et al. as having altered transcript levels during differentiation (67); information from additional studies is included in the third tab of Table S4 (8, 65, 68). These genes include, but are not limited to, the following: invariant surface glycoprotein genes (*ISG75*, Tb927.5.350, Tb927.5.360, Tb927.5.370, Tb927.5.380, Tb927.5.390, and Tb927.5.400); procyclin genes on chromosome 6, including the *GPEET2* gene (Tb927.6.510), *EP3-2* (Tb927.6.520), and procyclin-associated gene 3 (Tb927.6.530) (Fig. S6); expression site-associated genes (*ESAG1*, Tb927.4.1200, Tb927.10.105, *ESAG2*, Tb927.1.2040, *ESAG3*, Tb927.5.4600, Tb927.9.15940, *ESAG4*, Tb927.5.285b, *ESAG5*, Tb927.7.6860, Tb927.9.15890, *ESAG9*, Tb927.5.4620, Tb927.5.120, and Tb927.7.170 and *ESAG* pseudogenes Tb927.1.2060, Tb927.1.2070, Tb927.2.660, Tb927.2.910, Tb927.3.5790, Tb927.9.680, Tb927.9.16010, Tb927.9.16010, Tb927.9.16010, and Tb927.10.100), variant surface glycoprotein genes (*VSG*s), *COX* genes (Tb927.4.4620, Tb927.9.3170, Tb927.10.8320, and Tb927.11.13140), flagellar genes (Tb927.5.4480, Tb927.8.5440, Tb927.8.5460, and Tb927.8.5470), and adenylate cyclase genes (Tb927.11.1480, Tb927.5.320, Tb927.5.330, Tb927.6.270, Tb927.7.7470) (Table S4).

In conclusion, we have adapted the CUT&RUN technique for use in T. brucei parasites and used it to track *de novo* Bdf3 peak formation and changes in occupancy at Bdf3 binding sites during the transition from the bloodstream to the procyclic form. The mechanistic details for how changes in bromodomain protein occupancy might promote differentiation are an exciting area for future study.

## MATERIALS AND METHODS

### Parasite culture and strain generation.

Bloodstream parasites were cultured in HMI-9 at 37°C with 5% CO_2_. Differentiation was induced by resuspending parasites in differentiation medium (DTM) at 4 million cells/mL (69), adding 6 mM *cis*-aconitate, and incubating parasites at 27°C. Postdifferentiation parasites were maintained between 1 and 10 million cells/mL. The *BDF3*-HA/*BDF3* knockout (KO) strain was generated from EATRO 1125 AnTat1.1 90:13 (70). EATRO1125 Antat 1.1 lines were kept at densities below 600,000 cells/mL. The pMOTAG5H *BDF3*-HA construct (26) was linearized and introduced into parasites using an Amaxa Nucleofector kit. Correct integration was verified using PCR with the following primers: (i) HA rev (TATGGGTACGCGTAATCAGGCACA) and (ii) upstream Bdf3 5′ UTR for (TGTTGCAGGATATTGTGAGTGA). After this, the pyrFEKO *PAC*/GFP *BDF3* KO construct was linearized and transfected into Bdf3-HA-tagged parasites as described above and verified with the following primers: (i) GFP for (CTACAACAGCCACAAGGTCTAT) and (ii) downstream Bdf3 3′ UTR rev (AAACCGCAAAGTGATGAATGG). The control primers shown in figures are (i) Bdf3 3′ UTR for (CTTGTAGACAGCGGCATGGTTGG) and (ii) downstream Bdf3 3′ UTR rev (AAACCGCAAAGTGATGAATGG).

### CUT&RUN.

All spins prior to permeabilization were performed at 10°C and 2,738 × *g*. Spins after the permeabilization step occurred at 10°C and 4,602 × *g*. Fifty to 75 million parasites were harvested via centrifugation at 10°C and washed in 1 mL NP buffer containing 0.5 mM spermidine, 50 mM NaCl, 10 mM Tris-HCl (pH 7.5), and protease inhibitors. Parasites were spun and permeabilized using 100 μL NP buffer supplemented with 0.1% saponin (vol/vol) and 2 mM EDTA. Five micrograms of anti-HA (Sigma; H6908) or anti-IgG (Fisher; 02-6102) control antibody was added, and samples were rotated for 45 min at 25°C. For histone experiments, 1.5 μL rabbit anti-H3 (a kind gift from Christian Janzen) was added. Samples were washed and pelleted twice with 1 mL NP buffer. A volume corresponding to 1.4% of the sample was removed for flow cytometry analysis. Following the second wash, samples were resuspended in 100 μL NP buffer, and 0.5 μL protein A-micrococcal nuclease was added (a kind gift from Steven Henikoff). Samples were rotated for 5 min at 25°C. Samples were washed twice in 1 mL NP buffer as described above and resuspended in 100 μL NP buffer. CaCl_2_ was added to a final concentration of 2 mM, and nuclease digestion occurred for 5 min at 25°C. One hundred microliters of 2× STOP buffer (20 mM EDTA, 20 mM EGTA) with yeast spike-in DNA was immediately added. Samples were incubated for 10 min at 37°C to release insoluble nuclear chromatin. Samples were pelleted, and the supernatant containing the DNA was saved. SDS was added to a final concentration of 0.1%, proteinase K was added at 165 μg/mL, and RNase A was added to 6.5 μg/mL. Samples were incubated at 70°C for 10 min and purified using phenol chloroform or Ampure XP beads at 1.8× according to the manufacturer’s instructions.

### Flow cytometry.

All flow cytometry was performed on a Novocyte 2000R from Acea Biosciences (now Agilent). Parasites were resuspended in 100 μL HMI-9 and stained for 10 min on ice with mouse anti-rabbit IgG conjugated with phycoerythrin (PE) (Santa Cruz; sc-3753). Cells were washed twice in HMI-9 prior to analysis.

### Quantitative PCR analysis.

To quantify transcript levels of *PAD1*, parasites were grown to a density of 190,000 cells/mL for low-density samples or ≥1 million cells/mL for high-density samples. RNA was extracted from low- or high-density parasite populations using RNA Stat-60 (Tel-Test) following the manufacturer’s protocol and quantified on a NanoDrop 2000c. A 2.5-μg amount of RNA was used to generate cDNA using SuperScript IV VILO master mix (Fisher Scientific; 11756050) according to the manufacturer’s protocol. cDNA was amplified using 2× SYBR green master mix (Life Technologies 4309155) and primers and quantified on an Eppendorf Realplex2 instrument. For *PAD1*, the primers used were GACCAAAGGAACCTTCTTCCT and CACTGGCTCCCCTAAGCT, and for *URA3*, the primers used were CGGCAGCAGTTCTCGAGT and TGGCGTGTACCTTGAGGC. For differentiation experiments, the primers used for *EP1* were TCTGCTCGCTATTCTTCTGTTC and CCTTTGCCTCCCTTAGTAAGAC, and for *Tb927.10.9400 SF1*, the primers used were GGTATGGTTCATCAGGAGTTGG and CGTTAGCACTGGTATCCTTCAG.

### Generation of sequencing libraries.

Sequencing libraries were generated using the NEBNext Ultra II DNA library prep kit for Illumina (E7645) according to the manufacturer’s instructions, with the following modification: samples were incubated with USER enzyme immediately prior to PCR, rather than at an earlier step. NEBNext Multiplex oligonucleotides for Illumina were used to prepare multiplex samples (e.g., E7710).

### CUT&RUN sequencing analysis.

Sequencing was performed at the UCLA Technology Center for Genomics and Bioinformatics using an Illumina HISEQ 3000 with 50-bp single-end reads. CUT&RUN fastq files were trimmed using TrimGalore (http://www.bioinformatics.babraham.ac.uk/projects/trim_galore/) and aligned to the Tb927v5.1 genome using bowtie (71), and those requiring unique alignments used the following command: bowtie –best –strata -t -v 2 -a -m 1. Spike-in reads were aligned to the yeast sacCer3/R64 genome. MACS (42) was used in broad-peak mode to identify peaks of Bdf3 localization using an IgG control with the following arguments: -g 23650671, –keep-dup all, –nomodel, and –broad.

The DiffBind (48) package was used to identify regions with a change in Bdf3 occupancy. The GreyList ChIP package eliminated problematic regions from the analysis (0.66% of the genome). Four different normalization methods were then used to obtain normalized read counts for areas of interest determined by MACS: spike-in library size, spike-in RLE, background RLE (not using spike-in reads), and RPKM. Both RLE methods adjust for regional “background” read frequency by counting reads in nonoverlapping 15-kb genomic bins. This approach adjusts for broad patterns in background read enrichment while avoiding spurious adjustment to locally enriched regions. When MACS regions are used as input for the DiffBind program, it defines regions of interest as *x* bp upstream and downstream of the summit for the peak determined by MACS, where *x* is a variable determined by the user. We tested a number of *x* values and found similar results between 100 and 500 bp. The analysis presented here is performed with 200 bp on either side of the summit; thus regions of interest are each 400 bp long. DiffBind was used to identify regions with a statistically significant change in read counts (occupancy) at each time point using bloodstream values as a control with a cutoff of an adjusted *P* value (*P*_adj_) of <0.05 using the Benjamini-Hochberg adjustment to control the false-discovery rate (FDR) per the normalization technique. Once these regions were identified using each normalization method, a Venn diagram was used to identify “high-confidence” regions with changes in occupancy that were identified by all four normalization methods. To generate control regions, we randomly shuffled “high-confidence” regions for each chromosome using bedtools (56). Shuffled regions were maintained on the same chromosome, and peaks of Bdf3 localization identified by MACS were excluded. Control regions were run through the same DiffBind pipeline to obtain normalized read counts, which were plotted for each control region.

Overlap between ChIP and CUT&RUN-identified peaks was performed by first merging peaks that were within 5 kb in each data set and then identifying unique versus overlapping peaks using bedtools (56).

*De novo* peak formation was ascertained using a custom script. The program took as input the set of MACS-called Bdf3 consensus peaks at each time point and identified sites that were not called as Bdf3 peaks in bloodstream forms but subsequently were called as peaks at 2 consecutive time points following differentiation. The script for “disappearing” peaks took the same input and identified sites that were called as peaks in bloodstream parasites and not called as peaks at two consecutive time points following differentiation.

### Data availability.

Fastq files were deposited in the SRA database under project number PRJNA795567.
