# Supplementary figures and images for: Genomic Occupancy of the Bromodomain Protein Bdf3 Is Dynamic during Differentiation of African Trypanosomes from Bloodstream to Procyclic Forms
Source: mSphere. 2022 Jun 1;7(3):e00023-22. doi: 10.1128/msphere.00023-22 (PMC9241505; doi:10.1128/msphere.00023-22)

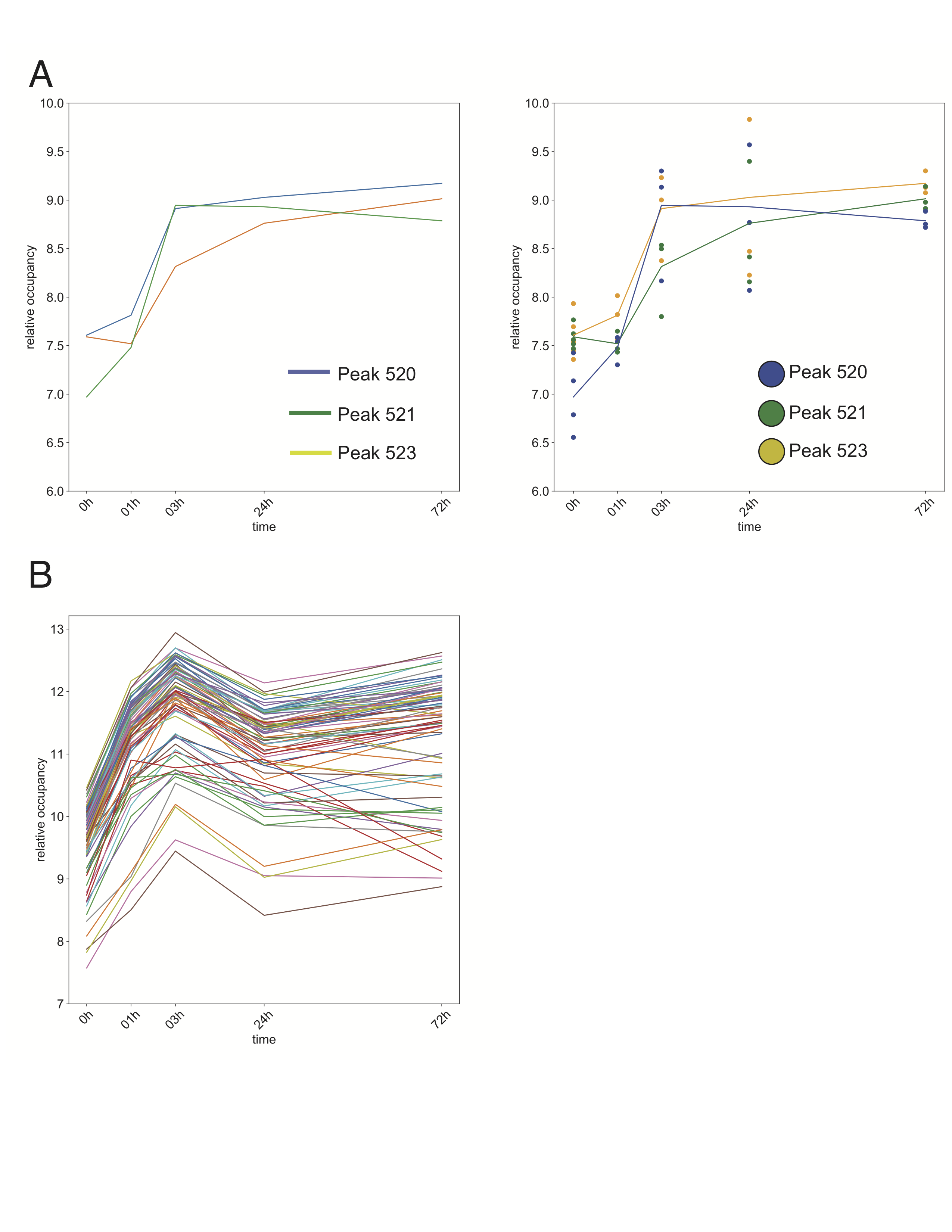

Supplement: FIG S1 [file msphere.00023-22-sf001.tiff]

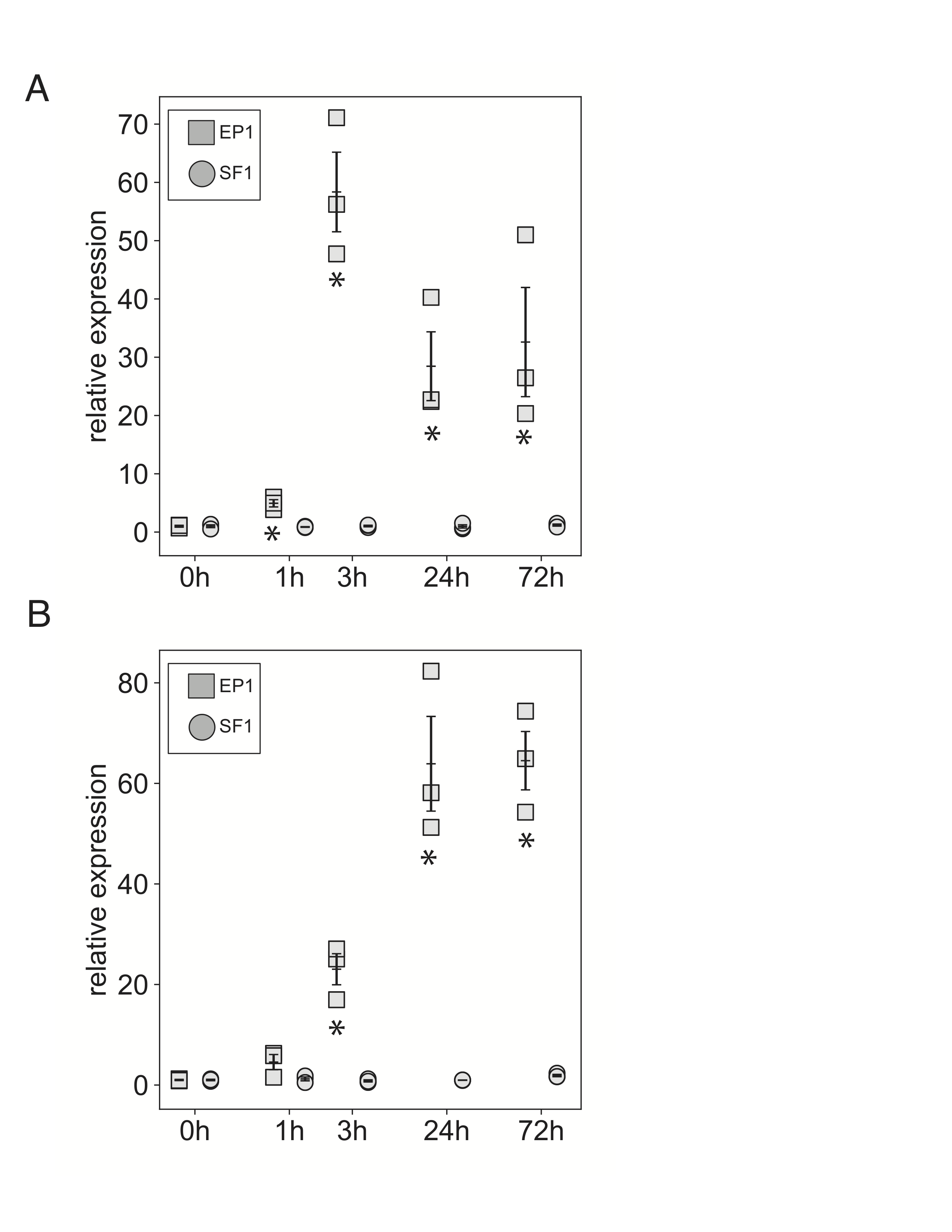

Supplement: FIG S2 [file msphere.00023-22-sf002.tif]

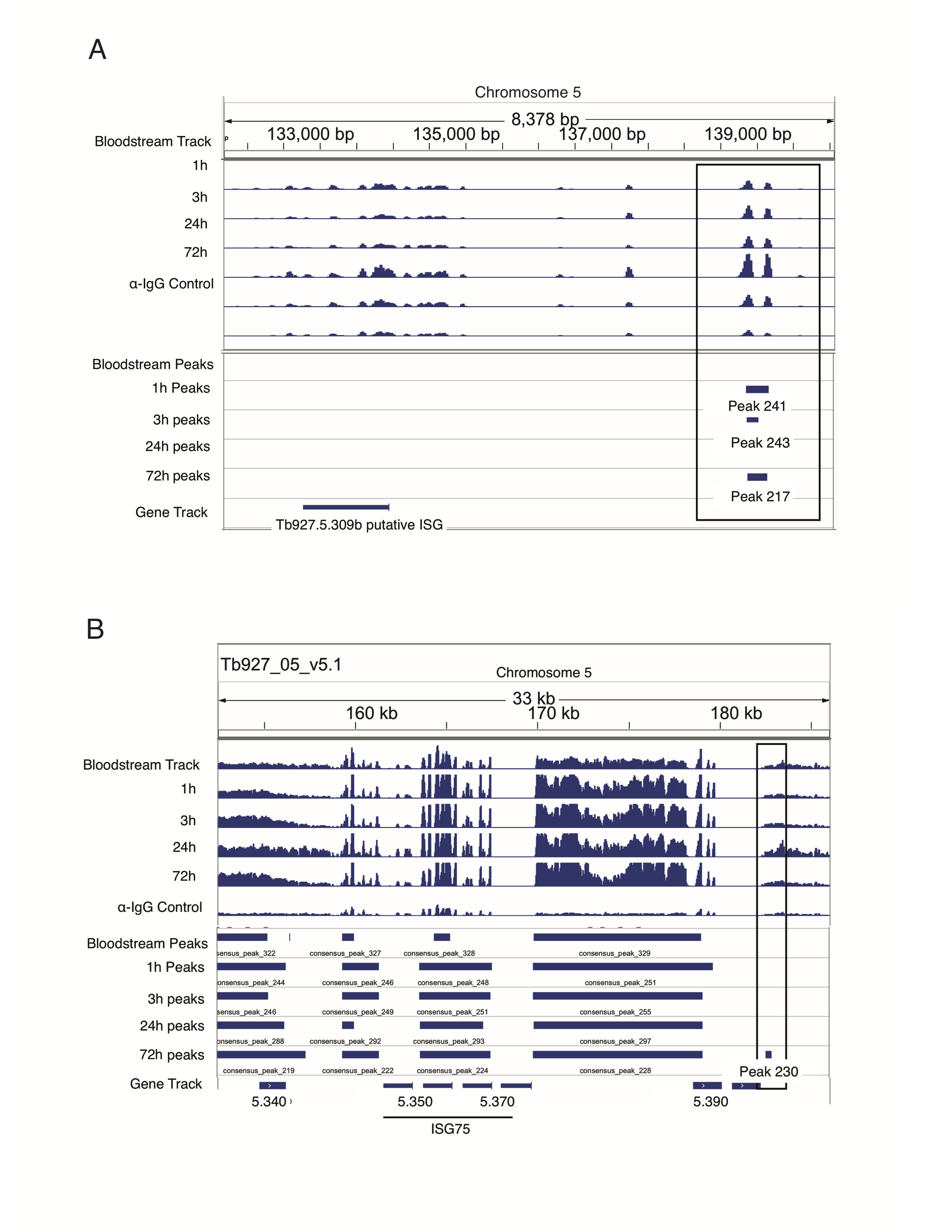

Supplement: FIG S3 [file msphere.00023-22-sf003.tif]

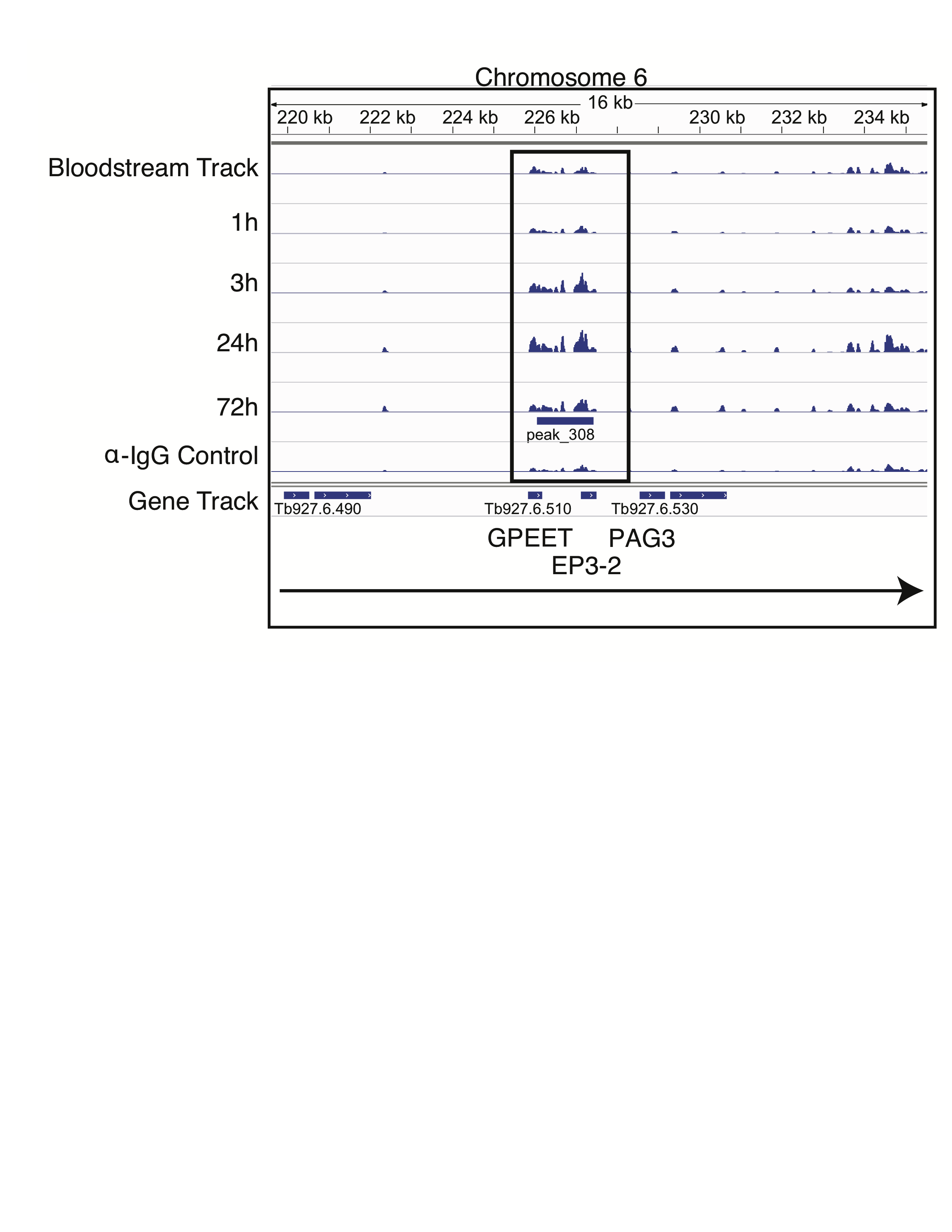

Supplement: FIG S6 [file msphere.00023-22-sf006.tif]

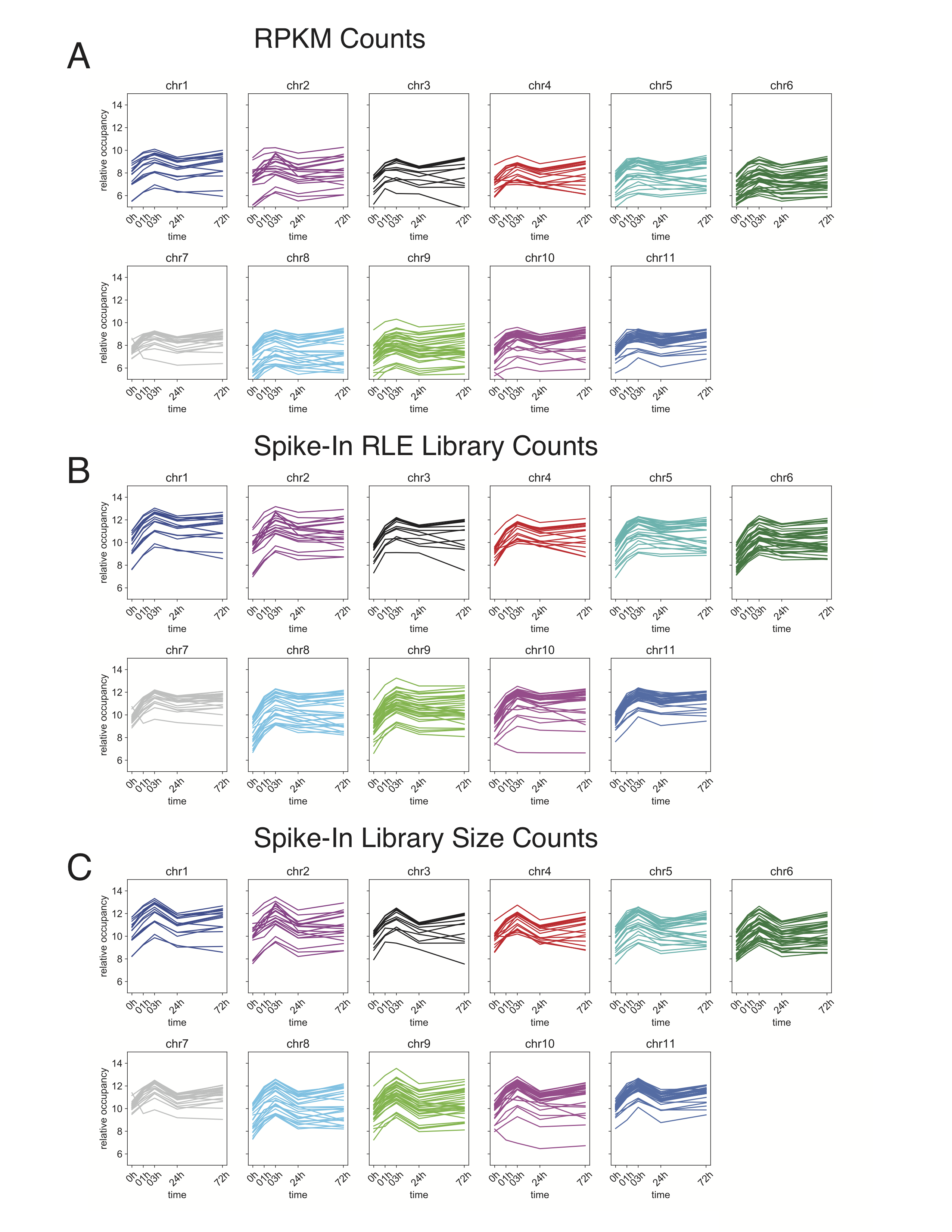

Supplement: FIG S4 [file msphere.00023-22-sf004.tiff]

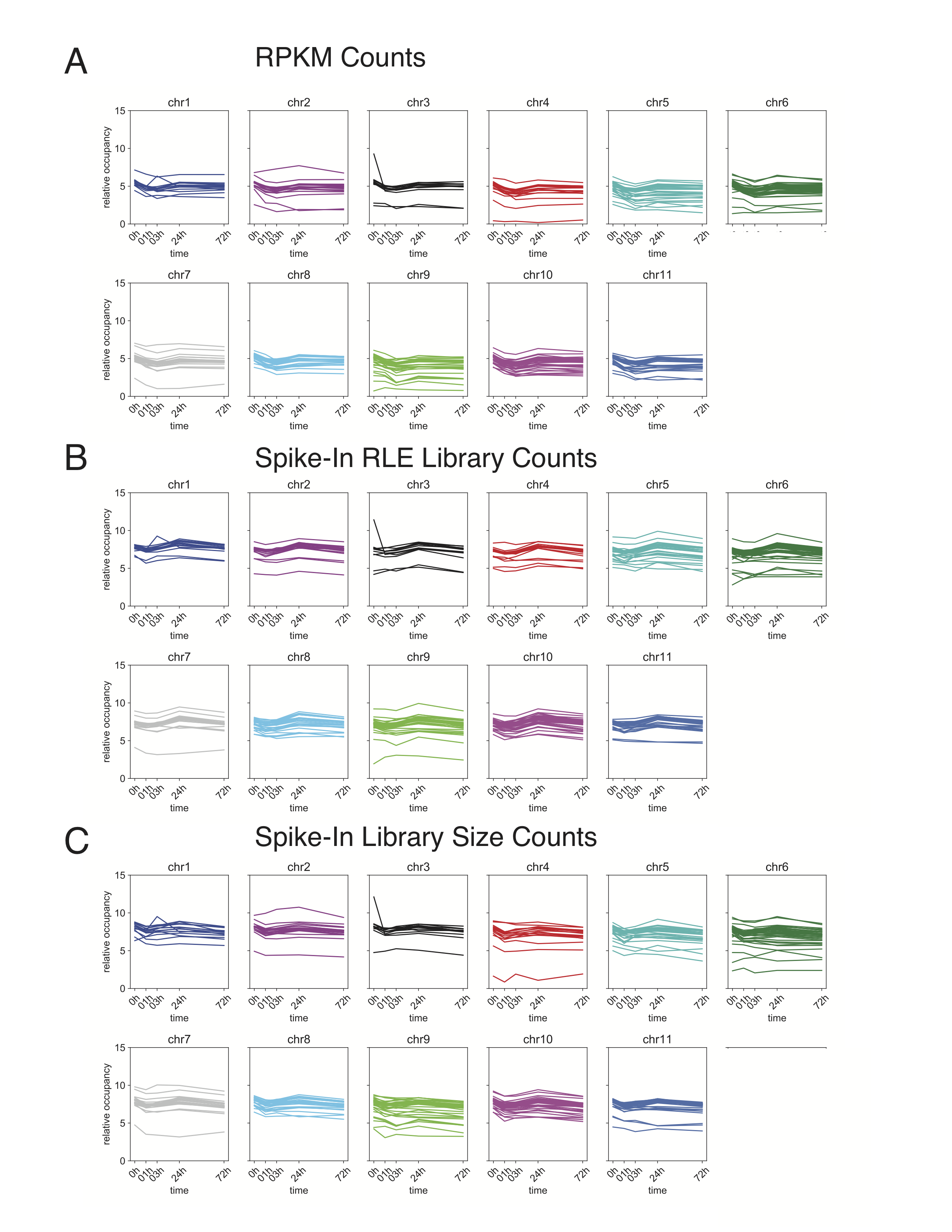

Supplement: FIG S5 [file msphere.00023-22-sf005.tiff]
